# Supplementary material for: Toxicity, Sublethal and Low Dose Effects of Imidacloprid and Deltamethrin on the Aphidophagous Predator Ceratomegilla undecimnotata (Coleoptera: Coccinellidae)
Source: Insects. 2021 Aug 3;12(8):696. doi: 10.3390/insects12080696 (PMC8396430; doi:10.3390/insects12080696)
Supplement: Supplementary file 1 [file insects-12-00696-s001.zip › insects-1273245-supplementary.pdf]

Toxicity, sublethal and low dose effects of imidacloprid and deltamethrin on the aphidophagous predator *Ceratomegilla undecimnotata* (Coleoptera: Coccinellidae)

Panagiotis J. Skouras[1*], Anastasios I. Darras[2], Marina Mprokaki[1], Vasilios Demopoulos[3], John T. Margaritopoulos[4], Costas Delis[2] and George J. Stathas[1]

[1.]Laboratory of Agricultural Entomology and Zoology, Department of Agriculture, University of the Peloponnese, Kalamata Campus, 24100 - Antikalamos, Greece

[2.]Department of Agriculture, University of the Peloponnese, Kalamata Campus, 24100 - Antikalamos, Greece

[3.]Laboratory of Plant Protection, Department of Agriculture, University of the Peloponnese, Kalamata Campus, 24100 - Antikalamos, Greece

[4.]Department of Plant Protection, Institute of Industrial and Fodder Crops, Hellenic Agricultural Organization "DEMETER" – NAGREF, Volos, Greece

Corresponding author: Panagiotis. J. Skouras
University of the Peloponnese,
Department of Agricultural Science,
Laboratory of Agricultural Entomology and Zoology
24 100 – Antikalamos, Kalamata, Greece
Telephone: +30 27210 45277
Mobile: +30 6948384430
Fax: +30 27210 45234
Email: pskouras@windowslive.com

Table S1 Total number and mortality of 4th instar larvae of *Ceratomegilla undecimnotata* by topical application of imidacloprid after 72-h.

Treatment (ng a.i. per insect)	Total number	72-h dead	Mortality (%)
0	40	0	0.0
5	40	1	2.5
10	40	3	7.5
20	40	12	30.0
40	40	23	57.5
80	40	32	80.0
160	40	39	97.5
320	40	40	100

Table S2 Total number and mortality of 4th instar larvae of *Ceratomegilla undecimnotata* by topical application of deltamethrin after 72-h.

Treatment (ng a.i. per insect)	Total number	72-h dead	Mortality (%)
0	40	0	0.0
0.13671875	40	0	0
0.2734375	40	1	2.5
0.546875	40	3	7.5
1.09375	40	6	15
2.1875	40	22	55
4.375	40	28	70
8.75	40	33	84.6
17.5	40	39	97.5
35	40	40	100
